# The impact of socioeconomic status on the utilization of total hip arthroplasty during 1995–2017: 104,055 THA cases and 520,275 population controls from national databases in Denmark

**DOI:** 10.1080/17453674.2020.1840111

**Published:** 2020-10-27

**Authors:** Nina M Edwards, Claus Varnum, Søren Overgaard, Alma B Pedersen

**Affiliations:** a Department of Clinical Epidemiology, Aarhus University Hospital, Aarhus N;; b Department of Orthopaedic Surgery, Lillebaelt Hospital, Vejle;;; c Department of Regional Health Research, University of Southern Denmark;; d Danish Hip Arthroplasty Register;; e Department of Orthopaedic Surgery and Traumatology, Odense University Hospital, Denmark;; f Department of Clinical Research, University of Southern Denmark, Denmark

## Abstract

Background and purpose — In Denmark, all citizens are guaranteed free access to medical care, which should minimize socioeconomic status (SES) inequalities. We examined the association between SES and the utilization of total hip arthroplasty (THA) by age and over time.

Patients and methods — Data on education, income, liquid assets, and occupation on 104,055 THA cases and 520,275 population controls were obtained from Danish health registers. We used logistic regression to estimate adjusted odds ratios (aOR) for THA with 95% confidence intervals (CI).

Results — Risk (CI) of THA was higher for 45–55-year-olds with lowest vs. highest education (aOR 1.4 [1.3–1.5]), and for those with lowest vs. highest income (aOR 1.1 [1.0–1.2]). The association between education and income and higher risk of THA decreased with increasing age. The risk of THA was lower for persons with lowest vs. highest liquid assets in all age groups and time periods. The risk of THA was higher for persons with lowest education in 1995–2000 (aOR 1.2 [1.1-1.3]), but diminished in 2013–2017 (aOR 1.0 [1.0–1.0]). For those on lowest income there was a higher risk of THA in 1995–2000 (aOR 1.2 [1.1–1.3]), changing to lower risk in 2013–2017 (aOR 0.8 [0.8–0.9]).

Interpretation — In a society where all citizens are guaranteed free access to medical care, we observed a social inequality in regard to the risk of THA with a development over time and in relation to age in most of our SES markers, showing a need for more patient involvement by implementing more focused interventions targeted to the most vulnerable patient groups identified as currently living alone, on low income, and with a low level of liquid assets.

In Denmark, the healthcare system provides tax-supported healthcare for all citizens, guaranteeing free access to medical care for emergency and hospital admission. In spite of this, inequality in healthcare has been found in Denmark in respect of socioeconomic status (SES) in stroke care, in chronic obstructive pulmonary disease, and in outcome among hip fracture patients (Tottenborg et al. 2016, Kristensen et al. 2017, Hyldgard et al. 2019).

Low SES is associated with a higher risk of developing hip osteoarthritis (OA) and a lower risk of seeking medical care even in countries with tax-based healthcare systems (Agerholm et al. 2013, Peltola and Järvelin 2014, Wetterholm et al. 2016). Thus, an increased need for total hip arthroplasty (THA) among individuals with low SES may be expected.

There are few studies regarding the association between SES and THA, and their results are contradictory. Some have found pro-rich-area inequality and lower rates of THA in patients of lower SES (Agabiti et al. 2007, Cookson et al. 2015, Wetterholm et al. 2016). Other studies have shown similar rates of THA across SES quintiles (Brennan et al. 2012). However, all these studies except Wetterholm et al. were based on group-level measures of SES or survey data. No previous study has examined the socioeconomic gradient in THA in Denmark, using individual-level register-based data and whether potential disparities are age- or time-dependent.

We conducted a population-based case-control study to examine the association between SES and the utilization of THA across different age groups and over time. We hypothesized that there is a socioeconomic inequality in THA utilization in Denmark.

## Patients and methods

### Setting

All Danish citizens are assigned a unique civil registration (CRP) number at birth, which is included in all Danish registers, allowing for unambiguous individual-level record linkage between the registers and enabling virtually complete long-term follow-up of all Danish inhabitants (Schmidt et al. 2014).

We used prospectively collected data from health registers, which encompassed the entire Danish population.

### Data sources

The Danish Civil Registration System (DCRS) contains information on the CPR number, vital and migrant status, cohabiting status, and municipality of residence (Schmidt et al. 2014).

The Danish Hip Arthroplasty Register (DHR) is a national clinical quality database established in 1995 (Gundtoft et al. 2016). The main variables are type of operation (primary or revision), operation side, primary diagnosis, and operation date (Gundtoft et al. 2016). Completeness of the DHR has been high from the beginning, being between 91% and 98% for primary THA (Gundtoft et al. 2016, Danish Hip Arthroplasty Register: Annual Reports 2019). In addition, the quality of registered data is high (Pedersen et al. 2004).

The Danish National Patient Registry (DNPR) is an administrative register covering all somatic admissions to all Danish hospitals since 1977 and outpatient and emergency room visits since 1995. Information reported to the DNPR includes admissions, diagnoses, treatments, and examinations. Diagnoses are reported to the DNPR according to the ICD-8 (from 1977 to 1993) or ICD-10 (starting in 1993) (Schmidt et al. 2015).

Statistics Denmark is a collection of register data that contains detailed individual-level information on socioeconomic characteristics on all Danish citizens. The Income Statistics Register includes information regarding family annual household income and liquid assets and the data are primarily supplied by tax authorities. The Population Education Register obtains information on the highest completed level of education and consists of data generated from administrative records of educational institutions and from surveys. The Register-based Labour Force Statistics (RAS) obtains a description of the affiliation with the labour market. The registers are administered by the Danish government and are updated yearly.

### Study population

We used the DHR to identify all patients over the age of 45 undergoing primary THA in Denmark from January 1, 1995 to December 31, 2017 and diagnosed with primary hip osteoarthritis (OA) (cases), using THA as a surrogate of the most severe hip OA cases. Only the first THA during 1995–2017 was included in the study cohort. The date of THA surgery was considered as the index date and the same date was used to identify the population controls over the age of 45 by matching for sex and region of residence on the index date. We used the DCRS to randomly select 5 population controls for each THA case.

### Socioeconomic status

For each THA case and population control we retrieved information on marital status, cohabitation, highest obtained education, family income, and occupation. In addition, SES was measured with family liquid assets on the index date. Highest obtained education was classified into 3 categories: low, defined as none or elementary school; medium, defined as more than elementary school, but less than university completed; and high, defined as university degree completed. Since a large proportion of the THA patients are senior citizens (> 65 years of age) with a state pension, family liquid assets was used to describe SES in patients > 65 years of age, whereas family income was used to assess SES in patients < 65 years of age (Robert and House 1996). This provides a more accurate estimate of overall socioeconomic stratification than using income and liquid assets through all age groups separately (Robert and House 1996). We obtained information on family income and liquid assets for the 5 years prior to surgery. To account for yearly variations in income and liquid assets, we calculated the average yearly total income and liquid assets in the 5 years prior to surgery for the patient and cohabiting partner. The family mean income and liquid assets were categorized into tertiles of increasing amount: low, medium, and high. Occupation was divided into the following categories: Director/chief executive, employer/self-employed, skilled worker, unskilled worker, unemployed, early retirement/pension, benefits and others (Table 1, Supplementary data). Because of low numbers, we regrouped occupation to include only retirement, working, and others in the age groups 76–85 and > 85.

**Table 7.** Demographic characteristics of total hip arthroplasty cases and population controls from the background population. Values are count (%) unless otherwise specified

	Cases	Controls
Factor	n = 104,055	n = 520,275
Female sex	58,422 (56)	292,100 (56)
Mean age (range)	69,7 (45–99)	62.3 (56–107)
Marital status		
Never married	6,541 (6)	55,931 (11)
Married	63,081 (61)	322,316 (62)
Divorced	12,085 (12)	72,684 (14)
Widow/widower	22,348 (21)	69,344 (13)
Cohabiting status		
Alone	33,143 (32)	146,802 (28)
Cohabitant	63,771 (61)	327,206 (63)
Other **^a^**	7,087 (7)	45,738 (9)
Education		
Low	43,247 (42)	175,473 (34)
Medium	36,964 (36)	212,530 (41)
High	15,788 (15)	98,134 (19)
Missing	8,036 (8)	34,138 (7)
Income, tertiles		
Low	46,310 (43)	159,376 (31)
Medium	38,526 (35)	172,510 (33)
High	24,086 (22)	188,183 (36)
Liquid assets, tertiles		
Low	31,674 (30)	163,356 (31)
Medium	33,394 (32)	166,125 (32)
High	35,645 (34)	168,505 (33)
Missing	3,342 (3)	22,289 (4)
Occupation		
Director/chief executive	6,577 (6)	29,437 (6)
Employer/self-employed	8,357 (8)	95,612 (18)
Skilled worker	7,835 (8)	85,295 (16)
Unskilled worker	2,286 (2)	22,793 (4)
Unemployed	1,125 (1)	15,408 (3)
Early retirement/pension	72,310 (69)	230,607 (44)
Benefits/public support	686 (1)	6,141 (1)
Other	4,777 (5)	34,940 (7)
Charlson comorbidity score		
Low	73,288 (70)	397,610 (77)
Medium	14,861 (14)	593,061 (11)
High	5,506 (5)	23,521 (4)

SD: Standard deviation.

**^a^** “Other” accounts for people coded as not having children living at home and people coded as households with multiple families.

### Covariates

Information on age and sex for both cases and controls was collected from the DCRS.

Comorbidities were obtained from the DNPR. We summarized the 10-year pre-surgery or pre-index-date hospital comorbidity history for each case and population control (Table 2, Supplementary data). We measured the comorbidity status by Charlson Comorbidity Index (CCI) score based on the 19 disease categories and defined 3 levels of the CCI: a score of 0 (low), given to patients with no known comorbidities included in the CCI; a score of 1–2 (medium); and a score of 3 or more (high) (Schmolders et al. 2015).

**Table 8.** Overall crude and adjusted odds ratios (OR) with 95% confidence intervals (CI) for THA during 1995–2017

	Unadjusted OR (CI)	Adjusted OR ^a^ (CI)
Marital status		
Never married	0.60 (0.58–0.61)	0.76 (0.74–0.79)
Married	1 (reference)	1 (reference)
Divorced	0.85 (0.83–0.87)	0.96 (0.93–0.99)
Widow/widower	1.65 (1.62–1.68)	0.89 (0.87–0.92)
Cohabiting status		
Alone	1.16 (1.14–1.18)	0.93 (0.91–0.96)
Cohabitant	1 (reference)	1 (reference)
Education, tertiles		
Low	1.53 (1.50–1.56)	1.08 (1.05–1.10)
Medium	1.08 (1.06–1.10)	1.00 (0.98–1.02)
High	1 (reference)	1 (reference)
Income, tertiles		
Low	2.19 (2.14–2.22)	1.18 (1.15–1.21)
Medium	1.72 (1.69–1.75)	1.19 (1.16–1.21)
High	1 (reference)	1 (reference)
Liquid assets, tertiles		
Low	0.92 (0.90–0.93)	0.73 (0.71–0.74)
Medium	0.95 (0.93–0.97)	0.87 (0.85–0.88)
High	1 (reference)	1 (reference)
Occupation		
Director/chief executive	1 (reference)	1 (reference)
Employer/self-employed	2.56 (2.47–2.65)	1.74 (1.68–1.81)
Skilled worker	1.05 (1.02–1.09)	1.07 (1.03–1.11)
Unskilled worker	1.20 (1.14–1.26)	1.17 (1.12–1.23)
Unemployed	0.84 (0.78–0.89)	0.94 (0.88 – 1.00)
Early retirement/pension	3.59 (3.50–3.67)	1.74 (1.68–1.79)
Benefits/public support	1.28 (1.18–1.39)	1.36 (1.25–1.48)
Other	1.56 (1.51–1.62)	1.31 (1.26–1.36)

**^a^** Adjusted for age, SES markers independently, calendar year, and CCI.

### Statistics

In order to describe the SES characteristics of cases and controls we calculated proportions of cases and controls with each specific marker, overall, and by age categories and year of surgery. Odds ratios (OR) with 95% confidence interval (CI) for THA according to each marker of SES were calculated using conditional logistic regression. We calculated crude odds ratios and odds ratios adjusted for age, SES markers independently, calendar year, and CCI. Sensitivity analyses were done when stratifying for age and calendar year.

The statistical analyses were performed in Stata version 15 (StataCorp, College Station TX, USA) and R version 3.6.1 (R Foundation for Statistical Computing, Vienna, Austria).

### Ethics, funding, and potential conflicts of interest

The study was approved by the Danish Data Protection Agency’s journal number 2015-57-0002 and Aarhus University’s journal number 2016-051-000001, record number 880. This study was reported following the STROBE and RECORD guidelines. We would like to acknowledge support from the Orthopaedic Research Fund, AP Møller Fund, and Aase and Ejnar Danielsens Fund. The funders had no role in the study design, data collection, and analysis, or in the preparation of the manuscript. The authors report no conflict of interest.

## Results

### Overall associations (Table 8)

Persons who never married had a lower risk of receiving a THA than patients who married (aOR 0.8 [CI 0.7–0.8]). The same association was seen in patients who were divorced (aOR 1.0 [CI 0.9–1.0]) or were widow/widower (aOR 0.9 [CI 0.9–0.9]). Persons who lived alone also had a lower risk of receiving a THA than cohabiting persons (aOR 0.9 [CI 0.9–1.0]).

Individuals with the lowest vs. highest level of education had a higher risk of receiving a THA (aOR 1.1 [CI 1.1–1.1]). The same was seen in those with the lowest vs. highest income (aOR 1.2 [CI 1.2–1.2]). The lowest vs. highest liquid asset was associated with lower risk of receiving a THA (aOR 0.7 [CI 0.7–0.7]). There was a higher risk of receiving a THA when the persons’ occupations were employer/self-employed, skilled worker, unskilled worker, early retirement/pension, and when receiving benefits/public support than being a director or chief executive.

## Discussion

In this large nationwide case-control study of 104,055 THA patients, we observed a substantial socioeconomic inequality in THA utilization. Married and cohabiting persons were at increased risk of receiving a THA. The association between low education, low income, and higher risk of THA was observed among patients 45–55 years of age, but decreased with increasing age. The inequality in the risk of THA by education decreased over calendar time, whereas the inequality by income and liquid assets was persistent.

### Cohabitation/social support

Our finding of the association between living alone or being divorced/never married/widow/widower and lower risk of THA could be due to those in need of THA often worrying about becoming reliant on family and friends for their daily activities (Mota et al. 2012), or just a lack of social and family support to assist in medical decision-making (Youm et al. 2015). There may also be a difference in willingness in regard to social support, where 1 study found OA patients to be less willing to undergo surgery if they were unmarried or beyond the age of retirement, which support our results (Mota et al. 2012).

### Education and income

Low education and lower income are associated with an increased OA severity and increased potential need for THA, as also found by Cleveland et al. (2013). It has been suggested that individuals with higher education are better able to process information regarding healthy behaviours and therefore may be able to postpone the need for a THA, equalizing the risk in the higher age groups (Brennan et al. 2012, Cleveland et al. 2013). However, was there supposed to be a greater disparity between level of education and income, and in the risk of THA, revealing a possibly greater unmet need for THA. (Cleveland et al. 2013, Wetterholm et al. 2016).

### Occupation and liquid assets

There is a clear dose–response relationship between occupational workload and increased risk of hip OA and therefore a greater risk of THA (Sun et al. 2019). This may explain some of our findings regarding occupation and higher risk of THA, when the patient’s occupation was employer/self-employed, skilled worker or unskilled worker in comparison with a potentially lower manual workload such as a position as a director or chief executive. Wetterholm et al. (2016) have shown similar results. This could further fit with our finding that lower liquid assets in persons above 65 years are a predictor of THA risk. Previous studies found that patients receiving benefits were twice as likely to require THA, but were also less likely to have had surgery (Ackerman and Busija 2012). Individuals who retire early or are on a pension have a higher risk of THA, but also show greater disease severity before THA (Dieppe et al. 2009).

### Time trends

Evaluation of time trends is important in order to ensure that the effort made by the Danish government to ensure equal access to healthcare irrespective of social position is working. Looking at the SES markers marital status and income, we found more inequality in the later years. In contradiction with this, Cookson et al. (2015) showed a pro-rich-area inequality in Europe, though the inequality was not significant in Denmark. However, their study has several limitations including missing data on the individual-level measures of SES, and their data on income was measured using sub-national administrative areas, which could cause misclassification of SES. In relation to education, we found a time trend from a wide spread of estimates in the years 1995–2000 to a narrowing of estimates in 2013–2017, indicating less social inequality in the latter years. Our study confirm that SES is a complex combination of an individual’s education, income, and occupation. These various SES markers may operate through different mechanisms to affect the risk of THA, including the potential for SES to influence lifestyle behaviours, preventive healthcare, and health management (Cleveland et al. 2013).

### Methodological considerations

**Figure 2.**
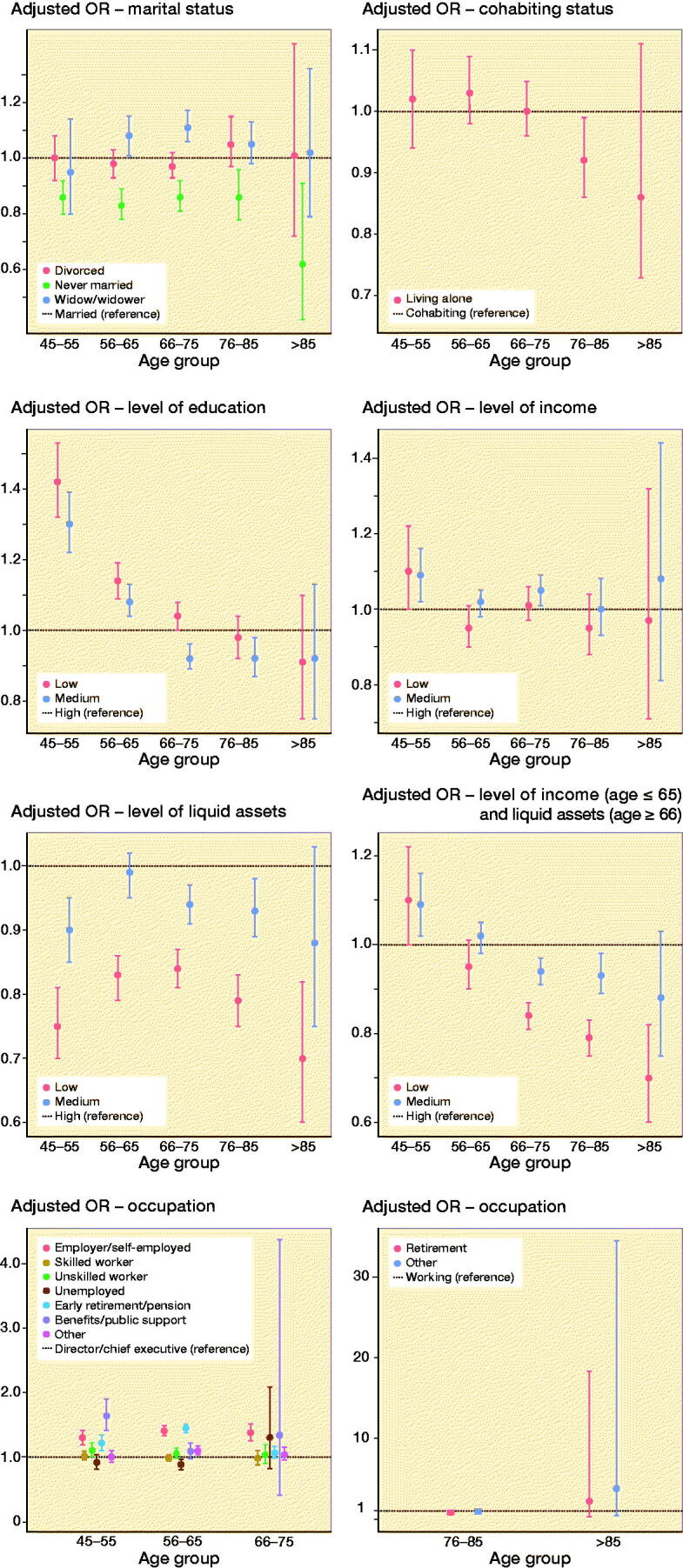
Dot plot for the adjusted OR with 95% confidence intervals, age stratified and adjusted for age, SES markers independently, calendar year, and CCI. Striped line: reference group.

Strengths of our study include the prospective data collection, where information on SES markers was collected from registers on an individual basis. The few missing SES data were distributed equally between cases and controls, and at random, which is why we believe that this at most could give an overestimation of our results. Unlike previous studies, we were able to include liquid assets as a SES marker providing a more accurate estimate of SES and prosperity for the individuals older than 65. This, however, introduces an apparent paradox where we see contradicting estimates between income and liquid assets. This limitation is considered when age stratifying the markers and joining them into Figure 2: level of income and liquid assets, thereby not pooling all ages in a variable where age has a major influence.

**Figure 3.**
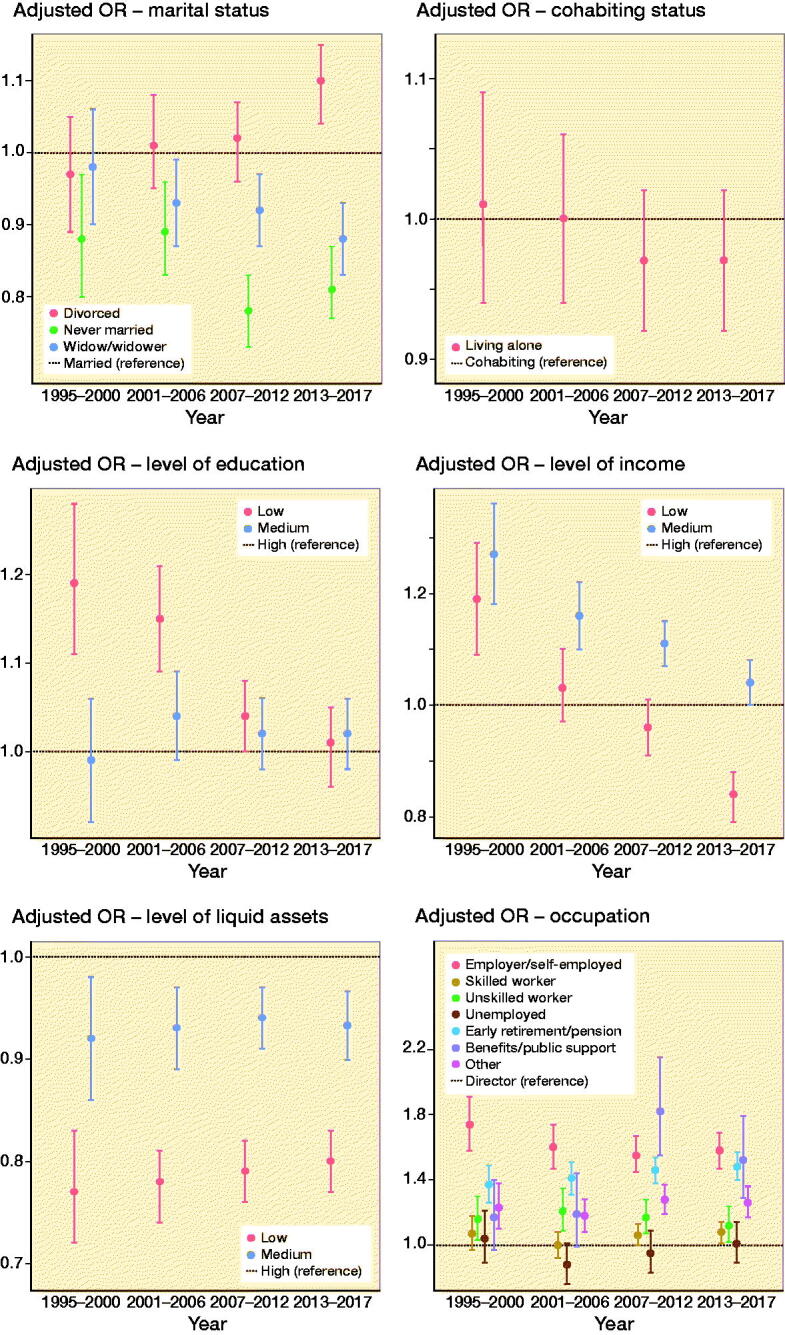
Dot plot for adjusted OR with 95% confidence intervals, stratified for calendar year and adjusted for age, SES markers independently, calendar year, and CCI. Striped line: reference group.

A limitation of our study is that we have no information regarding potential confounders such as lifestyle factors like smoking, alcohol, BMI, physical activity (Mota et al. 2012, Weiss et al. 2019). Another limitation is that the threshold for receiving a THA has changed with time and age possibly due to a change in surgeon-related factors as well as a change in the demands from society. This could influence our THA utilization in the age- and time-trend analysis. However, this does not have an impact on the distribution of cases in regard to the SES markers, leaving our results robust as regards this factor.

In conclusion, we found an age-dependent association between living alone, lower level of education, and lower level of income, and a high risk of THA. We also found that the inequality seen in the risk of THA by education decreased over time, suggesting a time trend towards less social inequality, while the inequality seen in the risk of THA by marital status and income increased over time. There is a development over time and in relation to age in most of our SES markers, showing a need for more patient involvement by implementing more focused interventions targeted to the most vulnerable patient groups identified as currently living alone, with low income and low level of liquid assets.

## Figures and Tables

**Figure 1.**
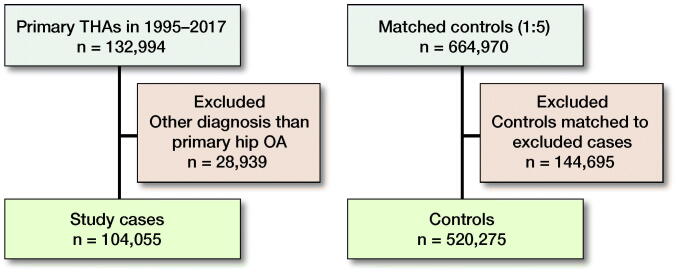
Flowchart of cases and controls.

## Description of the study population (Tables 3–6 in Supplementary data)

From 1995 to 2017, we identified 132,994 patients who received a THA and 664,970 population controls. We excluded 28,939 THA cases due to a diagnosis other than primary hip OA, as well as 144,695 corresponding matched population controls. The final study population included 104,055 THA cases and 520,275 controls (Figure 1). 56% of the study population were females. The mean age was 70 years (45–99) in the case group and 62 years (45–107) in the population control group. Further, cases had a lower education and a lower mean income, and there was a higher percentage of cases on early retirement or old-age pension (Table 7). Similar distribution is seen in the age- and year-stratified data.

## Age trends (Figure 2, Tables 9–11 in Supplementary data)

The reduced risk of receiving a THA among persons who were never married was seen in all age groups. Widows/widowers had a higher risk of receiving a THA than married persons in the age groups 56–65 and 66–75. The lower risk of receiving a THA when living alone was not consistent throughout the age groups.

Persons with the lowest level of education had a higher risk of receiving a THA in the age group 45–55 and 56–65; this was not the case in the age group > 85.

There was an association between lowest income and a higher risk of receiving a THA in the age group 45–55, but not in the age group 56–65+. The same decreasing age trend followed in the age group 66–75 with the lowest liquid assets, further decreasing the risk in the age group > 85.

In the age group 45–55, being an employer/self-employed was associated with a higher risk of receiving a THA than being a director/chief executive. This association was sustained throughout the age groups. Early retirement was associated with a higher risk of receiving a THA in all age groups under the age of 75, but not above 75 years.

## Time trends (Figure 3, Table 12 in Supplementary data)

Marital status showed a time-dependent association, moving from estimates in near proximity of each other in the years 1995–2000 to spreading and thereby more inequality in the years 2013–2017. There was a slightly reduced risk of receiving a THA in the years 1995–2000 when persons were divorced, but this association was time-dependent, changing to having a higher risk of receiving a THA in the years 2013–2017. The opposite time trend was seen for persons living alone.

Obtaining the lowest level of education was associated with a higher risk of receiving a THA in the years 1995–2000 than in the years 2013–2017. Likewise, the lowest income group was associated with a higher risk of receiving a THA than the highest income group in 1995–2000, but the difference vanished in the period 2013–2017.

The association between the lowest liquid assets and lower risk of receiving a THA was stable throughout the entire study period.

Patients who were an employer/self-employed were more likely to receive a THA in the years 1995–2000. This association decreased throughout the period in 2013–2017. Retiring early and receiving an old-age pension were associated with an increased risk of receiving a THA irrespective of the calendar year.

## Supplementary data

Tables1–6 and 9–12 are available as supplementary data in the online version of this article, http://dx.doi.org/10.1080/17453674.2020.1840111
